# MicroRNA-381 inhibits the metastasis of gastric cancer by targeting TMEM16A expression

**DOI:** 10.1186/s13046-017-0499-z

**Published:** 2017-02-13

**Authors:** Qinghua Cao, Fang Liu, Kaiyuan Ji, Ni Liu, Yuan He, Wenhui Zhang, Liantang Wang

**Affiliations:** 1 Department of Pathology, The first affiliated hospital of Sun Yat-sen University, Guangzhou, 510080 China; 2 Department of Oncology, Nanfang Hospital, Southern Medical University, Guangzhou, 510515 China; 3 Cancer Research Insitute, Southern Medical University, Guangzhou, 510515 China; 4 State Key Laboratory of Oncology in South China, Collaborative Innovation Center for Cancer Medicine and Department of Molecular Diagnostics, Sun Yat-sen University Cancer Center, Guangzhou, 510060 China

**Keywords:** MicroRNA-381, Gastric cancer, TMEM16A, TGF-β pathway, Epithelial-mesenchymal transition (EMT)

## Abstract

**Background:**

MicroRNA-381 (miR-381) has been reported to play suppressive or promoting roles in different malignancies. However, the expression level, biological function, and underlying mechanisms of miR-381 in gastric cancer remain poorly understood. Our previous study indicated that transmembrane protein 16A (TMEM16A) contributed to migration and invasion of gastric cancer and predicted poor prognosis. In this study, we found that miR-381 inhibited the metastasis of gastric cancer through targeting TMEM16A expression.

**Methods:**

MiR-381 expression was analyzed using bioinformatic software on open microarray datasets from the Gene Expression Omnibus (GEO) and confirmed by quantitative RT-PCR (qRT-PCR) in human gastric cancer tissues and cell lines. Cell proliferation was investigated using MTT and cell count assays, and cell migration and invasion abilities were evaluated by transwell assay. Xenograft nude mouse models were used to observe tumor growth and pulmonary metastasis. Luciferase reporter assay, western blot, enzyme-linked immunosorbent assay (ELISA) and immunohistochemistry were employed to explore the mechanisms of the effect of miR-381 on gastric cancer cells.

**Results:**

MiR-381 was significantly down-regulated in gastric cancer tissues and cell lines. Low expression of miR-381 was negatively related to lymph node metastasis, advanced tumor stage and poor prognosis. MiR-381 decreased gastric cancer cell proliferation, migration and invasion in vitro and in vivo. TMEM16A was identified as a direct target of miR-381 and the expression of miR-381 was inversely correlated with TMEM16A expression in gastric cancer tissues. Combination analysis of miR-381 and TMEM16A revealed the improved prognostic accuracy for gastric cancer patients. Moreover, miR-381 inhibited TGF-β signaling pathway and down-regulated epithelial–mesenchymal transition (EMT) phenotype partially by mediating TMEM16A.

**Conclusions:**

MiR-381 may function as a tumor suppressor by directly targeting TMEM16A and regulating TGF-β pathway and EMT process in the development of progression of gastric cancer. MiR-381/TMEM16A may be a novel therapeutic candidate target in gastric cancer treatment.

**Electronic supplementary material:**

The online version of this article (doi:10.1186/s13046-017-0499-z) contains supplementary material, which is available to authorized users.

## Background

Gastric cancer is the leading cause of cancer-related death worldwide, especially in China [1, 2]. Despite the treatments have been improved dramatically in recent years, invasion and metastasis, the major causes of gastric cancer related relapse and death, greatly impeded the treatment efficiency [3, 4]. However, the molecular mechanism underlying the invasion and migration of gastric cancer is still limited.

MicroRNA (miRNAs), a class of endogenous non-coding small RNAs, negatively regulate gene expression by binding to the 3′-untranslated regions (3′-UTR) of their target mRNAs, resulting in the degradation or translational repression [5, 6]. Emerging evidence has demonstrated that miRNAs are dysregulated in various human cancers and are associated with tumorigenic processes including cell proliferation, apoptosis, angiogenesis and invasion via their interaction with oncogenes and anti-oncogenes [5, 7]. Therefore, identifying specific miRNAs that play important roles in tumorigenesis would be beneficial for cancer diagnosis, prognosis, and therapy [8].

MicroRNA-381 (miR-381), located in a cluster within the 14q32.31 chromosomal region where miRNAs have been revealed to regulate cellular behaviors that are key to tumorigenicity [9, 10]. The expression of miR-381 is dysregulated in various cancer types. In lung adenocarcinoma [11], epithelial ovarian cancer [12], colon cancer [13, 14], breast cancer [15], hepatocellular carcinoma [16] and pituitary tumor [17], miR-381 is down-regulated and suppresses the malignancy of these tumors, suggesting that miR-381 may have potential roles as a tumor-suppressor miRNA. On the contrary, the expression of miR-381 is elevated in glioma [18, 19], synovial sarcoma [20], epitheliod sarcoma [21] and osteosarcoma [22], and silencing miR-381 inhibits the glioma growth [18] or increases the sensitivity of osteosarcoma cells to chemotherapeutic drugs [22]. Therefore, the functional roles of miR-381 in human cancers varied between different cancer types. However, little is known about the roles of miR-381 in the development of gastric cancer and the molecular mechanisms by which miR-381 exerts its functions.

TMEM16A (Transmembrane protein 16A), also known as ANO1, DOG1 or TAOS2, is a calcium-activated chloride channel [23] and plays a vital role in cell physiological behaviours, such as sensory transduction, epithelial secretion, smooth muscle contraction [24–26]. Accumulating evidence shows that TMEM16A is a candidate oncogene which plays crucial roles in the cellular events critical in tumorigenesis, including proliferation, apoptosis and metastasis [27–30]. Our previous study also reported that TMEM16A was highly expressed in gastric cancer and contributed to invasion and migration through transforming growth factor beta (TGF-β) signaling pathway, and TMEM16A overexpression was more pervasive than gene amplification in gastric cancer [31]. However, the upstream molecules regulating TMEM16A expression in cancer cells remain unclear. Recently, Mokutani et al. [32] demonstrates that ANO1 (TMEM16A) is a direct target of miR-132, and miR-132 overexpression markedly suppresses ANO1 expression level in colorectal cancer, suggesting that microRNAs may be involved in modulating TMEM16A expression.

In this study, we investigated the biological function and the molecular mechanism of miR-381 in gastric cancer. MiR-381 was notably decreased in gastric cancer clinical specimens and cell lines, and decreased expression of miR-381 was associated with adverse clinicopathological features and poor prognosis of gastric cancer patients. Functionally, miR-381 was found to inhibit the proliferation, migration and invasion of gastric cancer cells both in vitro and in vivo. Mechanically, miR-381 could suppress TGF-β signaling pathway and down-regulate EMT phenotype by targeting TMEM16A. Our findings elucidated the detailed roles of miR-381 in gastric cancer and further contribute to offering the effective therapeutic targets for the treatment of gastric cancer.

## Methods

### MicroRNA expression profile data from Gene Expression Omnibus (GEO)

MicroRNA array expression profile data GSE26595 and GSE28700 were downloaded from open Gene Expression Omnibus (GEO) database (https://www.ncbi.nlm.nih.gov/geo/), which contained 60 primary gastric cancer tissues and 8 surrounding non-cancer tissues, 22 gastric cancer tissues and paired normal tissues, respectively. These microRNA array expression data were analyzed by the Qlucore Omics Explorer (QOE 3.1) bioinformatics software (http://www.qlucore.com/). The QOE offers state-of-the-art mathematical and statistical methods, and its main features are the ease of use and speed with which datasets can be analyzed and explored [33, 34]. The miR-381 expression level were explored in primary gastric cancer tissues and paired non-tumor tissues.

### Patients and tissue specimens

Paraffin-embedded pathological specimens from 103 primary gastric cancers and paired adjacent non-tumor tissues were obtained from the archives of the Department of pathology, the First Affiliated Hospital of Sun Yat-Sen University, Guangzhou, China, between July 2006 and June 2011. None had received preoperative radiotherapy or chemotherapy before surgery. Postsurgical chemotherapies were performed depending on the severity of the disease and according to the National Comprehensive Cancer Network (NCCN) guidelines. The clinical and pathological parameters are shown in Table 1. The patients’ TNM stage was defined according to AJCC staging system for gastric cancer [35]. All the samples were collected with patient’s informed consent after approval from the Institute Research Medical Ethics Committee of the First Affiated Hospital, Sun Yat-sen University.

**Table 1:** Correlation of miR-381 expression with clinicopathological parameters

Variable	All Cases	miR-381 expression		*P* value^a^
		High	Low	
Gender				
Male	71	43	28	0.829
Female	32	18	14	
Age at surgery				
<57^b^	59	34	25	0.840
≥57	44	27	17	
Tumor size				
≥5 cm	44	24	20	0.425
<5 cm	59	37	22	
Histological type				
Intestinal	88	51	37	0.583
Diffuse	15	10	5	
TNM				
I + II	54	43	11	**0.000**
III + IV	49	18	31	
Lymph node metastases				
Present	59	27	32	**0.002**
Absent	44	34	10	

^a^Chi square test; ^b^median age

The entries in boldface with significance as *P* < 0.05

### Cell lines and transfection

Six human gastric adenocarcinoma cell lines, AGS, MKN-45, MKN-28, SGC-7901, BGC-823, MGC-803 and one human gastric epithelial cell line GES-1, were used in this study. All cell lines were obtained from Institute of Biochemistry and Cell Biology at the Chinese Academy of Sciences (Shanghai, China), and were grown in F-12 k (ATCC) supplemented with 10% fetal bovine serum and 1% penicillin-streptomycin at 37 °C with humidified 5% CO_2._

2 × 10^5^ cells were plated in 6-well plates and transfected with 100 nmol/L miRNAs employing Lipofectamine RNAiMAX Transfection Reagent (Invitrogen) according to the manufacturer’s protocol. The agomiR miR-381 (agomiR-381), antagomiR miR-381 (antagomiR-381) and their negative control (Con) Oligonucleotides were purchased from Shanghai GenePharma Co. Ltd. The coding sequences TMEM16A were amplified by PCR and inserted into pcDNA3.1 vector (Invitrogen) to generate TMEM16A overexpression vectors.

### RNA extract and quantitative real-time PCR

Total RNA was extracted from gastric cancer cell lines, tumor tissues and paired adjacent non-tumor tissues using Trizol Reagent (In vitrogen, USA) according to the manufacturer’s instructions. RNA was reverse transcribed to cDNA by using One Step PrimeScript miRNA cDNA Synthesis Kit (TaKaRa), and quantitative real time PCR was performed using SYBR Premix Ex Taq II (TaKaRa). MiRNA expression levels were normalized against the endogenous U6 small nuclear RNA (U6 snRN A) control. The relative expression level of miR-381 in each matched cancer and adjacent non- tumor tissue was calculated by the 2^-ΔΔCT^ method. The sequences of the PCR primers were as follows: miR-381 forward, 5′-AGTCTATACAAGGGCAAGCTCTC-3′, and reverse primer was Uni-miR qPCR primer (TaKaRa); U6 forward, 5′-CTCGCTTCGGCAGCACA-3′ and reverse, 5′-AACGCTTCACGAATTTGCGT -3′; TMEM16A forward, 5′-ATTTCACCAATCTTGTCTCCATCA-3′, and reverse, 5′-TGATAACTCCAAGAACGATTGCA-3′; GAPDH forward, 5′CTCCTCCTGTTC GACAGTCAGC-3′, and reverse 5′-CCCAATACGACCAAATCCGTT-3′.

### In vitro cell proliferation assay

Cell count and MTT assay was used to determine the cell proliferation capacity. For cell count, cells were serum free for 24 h. Then cells were trypsinized and equal number (2 × 10^5^) of cells from each group was plated into 6-well culture plates in complete culture medium for 0, 1, 2, 3, and 4 days. For MTT assay, cells were serum free for 24 h. Then cells were plated in 96-well plates at 2000 per well in a final volume of 100 μl. Then at 0, 1, 2, 3, and 4 days, 25 μl of MTT stock solution was added to each well and incubated for 4 h. The absorbance was measured at 570 nm. The assays were performed in triplicates.

### In vitro cell migration and invasion assays

Cell migration and invasion assays were performed using Transwell chambers with or without a Matrigel (BD Biosciences) coating. Briefly, 2 × 10^4^ transfected cells in serum-free DMEM medium were placed into the upper compartment of the chamber. Medium containing 10% FBS were added to the lower chamber to serve as chemoattractant. After incubation for 48 h in a humidified atmosphere of 5% CO_2_ at 37 °C, the cells on the upper surface of the filters were removed from the top well with a cotton swab, while the cells migrated or invaded into the the lower surface of the filters were fixed with 70% methanol for 30 min and stained with 0.2% crystal violet for 10 min. Photographs of 5 randomly selected fileds of the fixed cells were taken and counted under a light microscope at the magnification of 100×.

### In vivo tumor formation and metastasis assays

Animal experiments were performed in compliance with the guidelines for the Welfare of Experimental Animals in Sun Yat-sen University. For in vivo tumorigenicity assay, briefly, 5 × 10^5^ agomiR-381 and negative control transfected cells were subcutaneously into the right flank of each nude mouse, of 4- to 5-week old nude mice (5 mice per group). Tumor volume was measured every 3 days over a 3-week period (formula: tumor volume (mm^3^) = length × width^2^ × 0.5). For in vivo metastasis assay, briefly, 5 × 10^5^ cells transfected with agomiR-381 or negative control were intravenously injected through the tail vein of 4- to 5-week-old nude mice (5 mice per group). After 4 weeks, the mice were euthanized and the number of metastases per lung was determined under a dissecting microscope. The lungs were excised and embedded in paraffin. Then, hematoxylin and eosin (H&E) staining was performed to affirm the presence of tumors.

### Luciferase reporter assay

The wild-type TMEM16A-3′UTR (WT) and mutant TMEM16A-3′UTR (MUT) containing the putative binding site of miR-381 were chemically synthesized and cloned into the downstream of the firefly luciferase gene in a pGL3-promoter vector (Ambion). The gastric cancer cells were seeded in 24-well plates for 24 h, and then were co-transfected with wide-type or mutant-type TMEM16A vector, and agomiR-381 or negative control. After 48 h, cells were harvested, the activities of both firefly and Renilla luciferases in cell lysates were measured using the Dual Luciferase Reporter Assay System (Promega). Renilla luciferase was used for normalization.

### Western blot assay

Cells were collected and lysed with the RIPA buffer containing protease inhibitor. Protein concentration was determined by the Bradford method with bovine serum albumin as the control. Equal amounts of protein lysates (30 μg each lane) were separated 10% SDS-PAGE gel and then electrotransferred to polyvinylidene difloride membranes. The membranes were then blocked and incubated with primary antibodies against TMEM16A (1:500, Abcam), β-actin antibody (1:1000, Santa Cruz) respectively, for 2 h at room temperature, and then incubated with appropriate horseradish peroxidaseconjugated secondary antibodies (1:1000, Cell Signaling Technology) for 1 h at room temperature. Final detection was carried out with LumiGLO chemiluminescent reagent (New England Biolabs) as described by the manufacturer. The densities of target bands was accurately determined by the computer-aided 1-D gel analysis system.

### Immunohistochemistry (IHC)

The paraffin-embedded tissue sections (4 μm in thickness) were subjected to IHC assays as previously described [31].

### Enzyme-linked Immunosorbent Assay (ELISA)

Sandwich ELISA using Quantikine human TGF-β1 immunoassay and TGF-β2 immunoassays (R&D Systems) to detect TGF-β1 and TGF-β2 levels as described previously [31].

### Statistical analysis

Statistical analysis was performed using SPSS standard version 19.0 and GraphPad Prism 5. Student’s *t* test was used to compare the levels of cellular proliferation, migration and invasion between different groups. Chi-square test was used to compare the levels of miR-381 expression and various clinicopathological parameters of gastric cancer patients. Survival curves calculation and overall survival (OS)/progression-free survival (PFS) curve plotting used the Kaplan-Meier method, and the Log-Rank test was applied to compare the distribution between patient subsets. *P* < 0.05 was set to be statistically significant.

## Results

### MiR-381 is decreased in gastric cancer tissues and cells

**Fig. 1:**
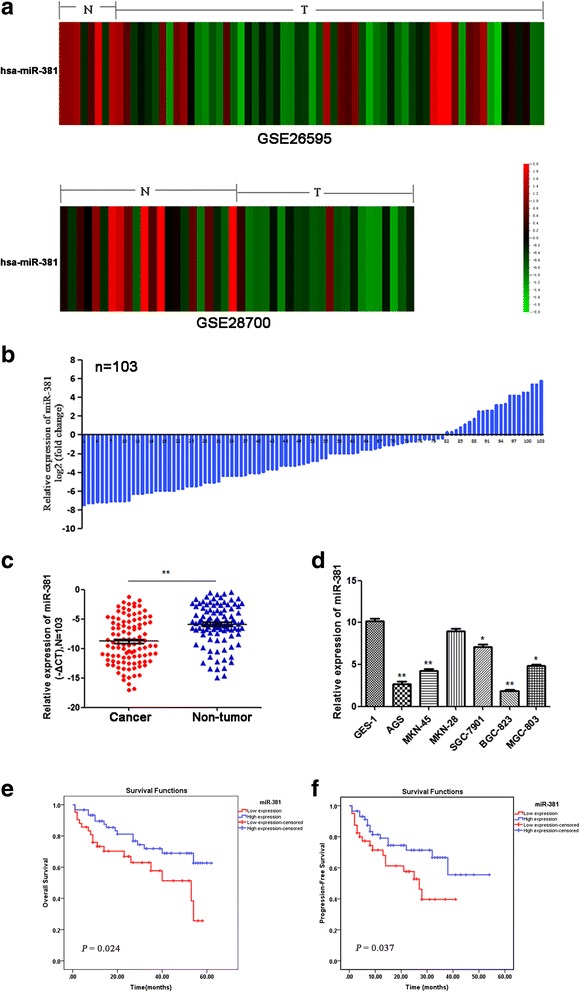
MiR-381 is low-expressed in gastric cancer tissues and cell lines. **a** Bioinformatic analysis of the level of miR-381 expression of GSE26595 and GSE28700 datasets showed that miR-381 was significantly lower in gastric cancer tumors (T) than in non-tumor tissues (N). (*P* < 0.01, *P* < 0.01, respectively); **b** QRT-PCR analysis of miR-381 expression in 103 pairs gastric cancer tissues and their corresponding adjacent non-tumor tissues. The expression of miRNA was normalized to U6 snRNA; **c** Relative miR-381 expression levels in gastric cancer tissues and adjacent non-tumor tissues. **P < 0.01; **d** QRT-PCR analysis of miR-381 expression in gastric cancer cell lines (AGS, MKN-45, MKN-28, SGC-7901, BGC-823, MGC-803) and gastric epithelial cell line (GES-1). *P < 0.05, **P < 0.01; **e**, **f** Overall survival and progression-free survival were compared between gastric cancer patients with low expression level of miR-381 and those with high level of miR-381

To explore the expression pattern of miR-381 in gastric cancer, we first downloaded microRNA array expression profile datasets GSE26595 and GSE28700 from the open Gene Expression Omnibus (GEO) database. QOE3.1 software was used to analyze the expression of miR-381 in gastric cancer tissues and adjacent non-tumor tissues. The results showed that miR-381 was significantly down-regulated in gastric cancer tissues compared with adjacent non-tumor tissues in both GSE26595 and GSE28700 (Fig. 1a) (*P* < 0.01, *P* < 0.01, respectively). To further validate the findings, we detected miR-381 in 103 paraffin embedded gastric cancer tissues and paired adjacent non-tumor tissues through qRT-PCR. As expected, down-regulation of miR-381 was observed in 81 (78.6%) cases of gastric cancer tissues, which was markedly lower than that in adjacent non-tumor tissues (Fig. 1b and c). In cell level, miR-381 expression was decreased in AGS, SGC-7901, BGC-823, MKN-45 cell lines than that in the normal gastric epithelial cell line GES-1 (Fig. 1d). All the above results indicated that miR-381 was down-regulated in gastric cancer.

### Down-regulation of miR-381 is associated with adverse clinicopathological features and poor prognosis

We next examined the potential clinical significance of miR-381 in gastric cancer. Based on relative expression in cancer/adjacent non-tumor < or >0.5, the 103 gastric cancer cases were divided into two groups: the miR-381 high expression group (*n* = 41) and the miR-381 low expression (*n* = 62). The correlation between miR-381 expression and clinicopathological characteristics was shown in Table 1. Low miR-381 expression was positively associated with present lymph node metastasis, advanced tumor stage (*P* = 0.002, *P* = 0.000, respectively). However, the expression level of miR-381 was not significantly associated with gender, age at surgery, tumor size, histological type.

Kaplan–Meier survival analyses showed that gastric cancer patients with low miR-381 expression had a significantly shorter overall survival and progression-free survival time than those patients with high miR-381 expression (*P* = 0.024, *P* = 0.037, respectively) (Fig. 1e and f). However, multivariate cox regression analysis failed to identify miR-381 expression as an independent prognostic factor for gastric cancer patients (data not shown). Based on these findings, we speculated that miR-381 might play a crucial role in gastric cancer development.

### MiR-381 inhibits gastric cancer cell proliferation, invasion and migration in vitro

**Fig. 2:**
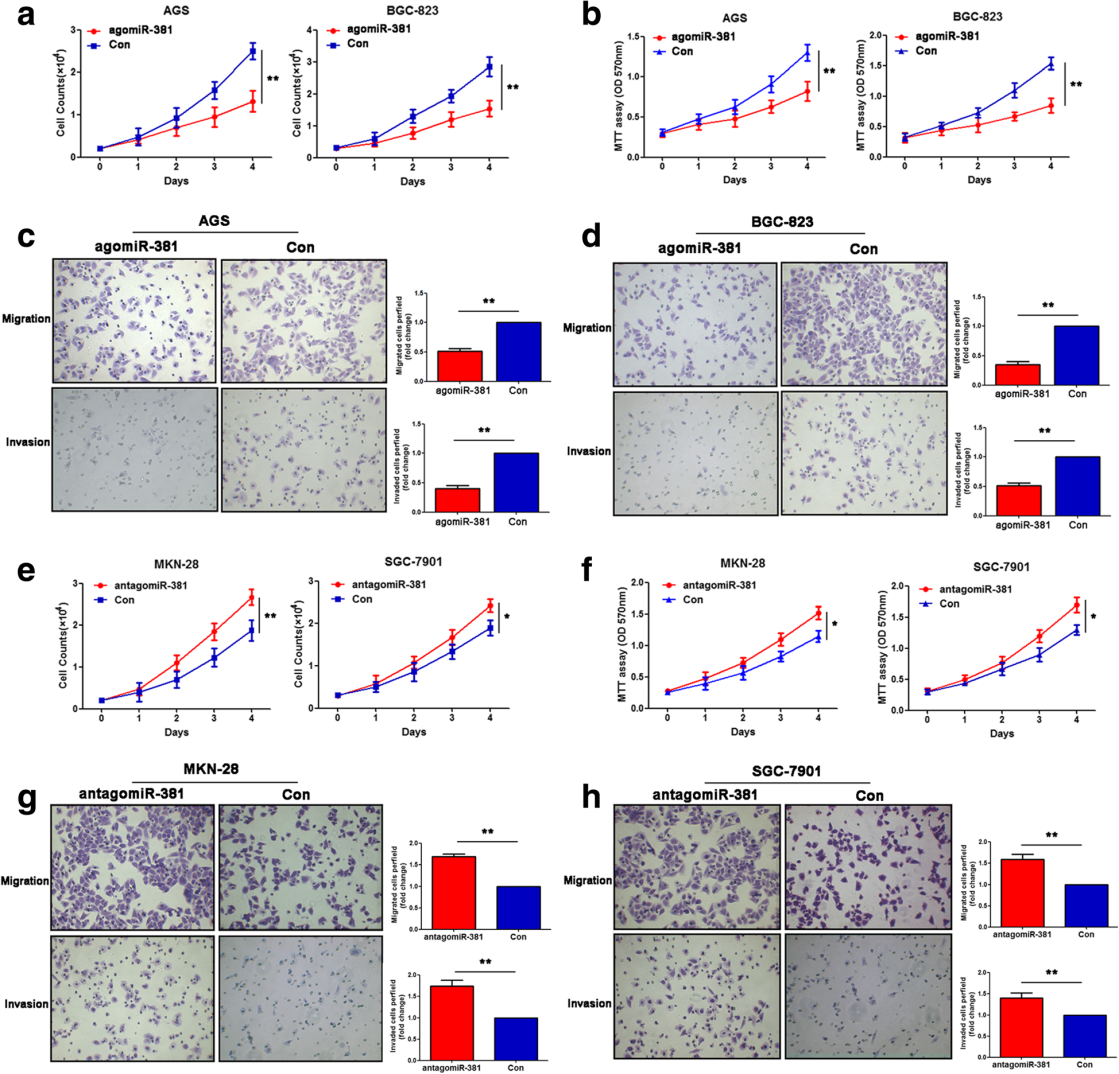
MiR-381 suppresses gastric cancer proliferation, migration and invasion in vitro. **a**, **b** Cell count and MTT assay analysis were used to evaluate the proliferation of gastric cancer cells (AGS and BGC-823) after transfection with the miR-381 agomiR (agomiR-381) or negative control (Con). **P < 0.01. **c**, **d** Transwell assay was performed to detect the ability of migration and invasion of agomiR-381 transfected gastric cancer cells and their negative control. **P < 0.01. (**e**, **f**) Cell count and MTT assay analysis were used to evaluate the proliferation of gastric cancer cells (MKN-28 and SGC-7901) after transfection with the miR-381 antagomiR (antagomiR-381) or negative control (Con). *P < 0.05, **P < 0.01. **g**, **h** Transwell assay was performed to detect the ability of migration and invasion of antagomiR-381 transfected gastric cancer cells and their negative control. **P < 0.01

To investigate the biological function of miR-381 in development and progression of gastric cancer, we performed gain- and loss- function experiments through transfection with agomiR-381 and antagomiR-381. First, AGS and BGC-823 cells, which were lower expression of miR-381, were transfected with agomiR-381. Ectopic expression of miR-381 of the two gastric cancer cell lines was confirmed by qRT-PCR after transfection (Additional file 1: Figure S1). Cell count and MTT assay showed that the cancer cells proliferation was dramatically inhibited in miR-381 overexpression group compared to that in the negative control group (Fig. 2a and b). In order to investigate the role of miR-381 in cell migration and invasion, transwell chamber assay was performed in gastric cancer cells. We found ectopic expression of miR-381 in AGS and BGC-823 cells could significantly inhibit cell invasion and migration. The number of invasive and migrated cells in the miR-381 ectopic expression group was notably decreased compared with the negative control group in two gastric cancer cell lines (Fig. 2c and d). On the other hand, we transfected MKN-28 and SGC-7901 which expressed relative higher levels of miR-381 using antagomiR-381. QTR-PCR was used to comfirmed the decrease expression of miR-381 (Additional file 2: Figure S2). As expected, inhibition of miR-381 markedly faciliated the proliferation, migration and invasion of MKN-28 and SGC-7901 cells (Fig. 2e-h). These results proved that miR-381 inhibited proliferation, invasion and migration of gastric cancer cells in vitro.

### Overexpression of miR-381 inhibits tumor growth and metastasis in vivo

**Fig. 3:**
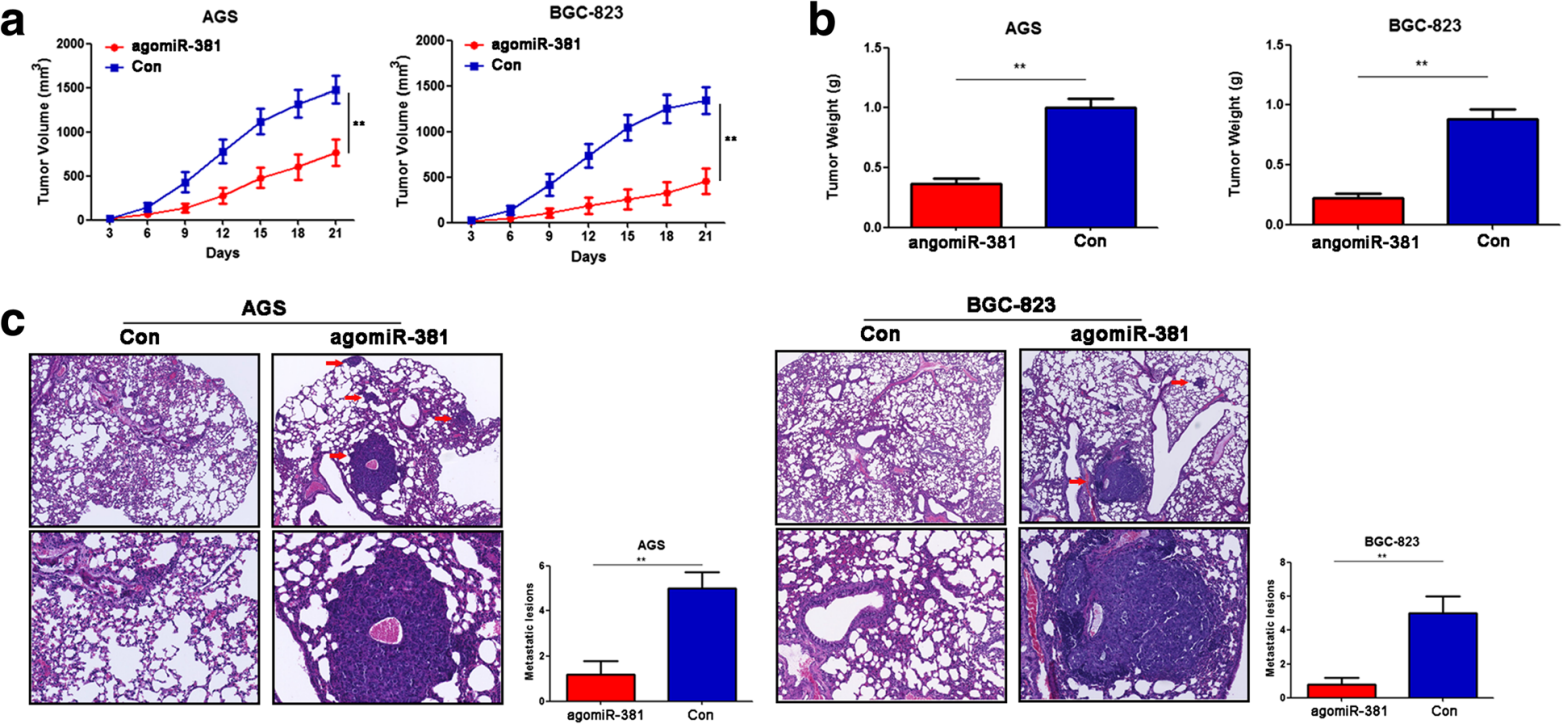
Overexpression of miR-381 inhibits gastric cancer growth and metastasis in vivo. **a** Growth curve of tumors in nude mice. Tumor diameters were measured every 3 days. **P < 0.01. **b** The average weight of tumors in nude mice.**P < 0.01. **c** Representative HE staining of lung metastasis of agomiR-381 and negative control group. **P < 0.01. Red arrows show the position of lung metastasis

Given that miR-381 inhibited the proliferation, migration and invasion of gastric cancer cell in vitro, we further detected the influence of miR-381 on tumor growth and metastasis in vivo. The in vivo role of miR-381 in tumor growth was evaluated by xenograft tumor formation in athymic nude mice. The tumor growth curve indicated that tumors in miR-381 overexpression group grew much more slowly than tumors in the negative control group (Fig. 3a). Moreover, overexpression of miR-381 can lead to significantly reduced tumor weight to the negative control group mice (Fig. 3b). To evaluate the in vivo effects of miR-381 on tumor metastasis, nude mice were injected intravenously in the tail vein with miR-381 overexpression or negative control gastric cancer cells respectively. Histological analysis revealed that the number of metastatic nodules was significantly reduced in the lung of mice injected with miR-381 overexpression cells compared to that with negative control cells (Fig. 3c). Taken together, these data indicated that miR-381 inhibited growth and metastasis of gastric cancer cells in vivo.

### TMEM16A is a direct target of miR-381

**Fig. 4:**
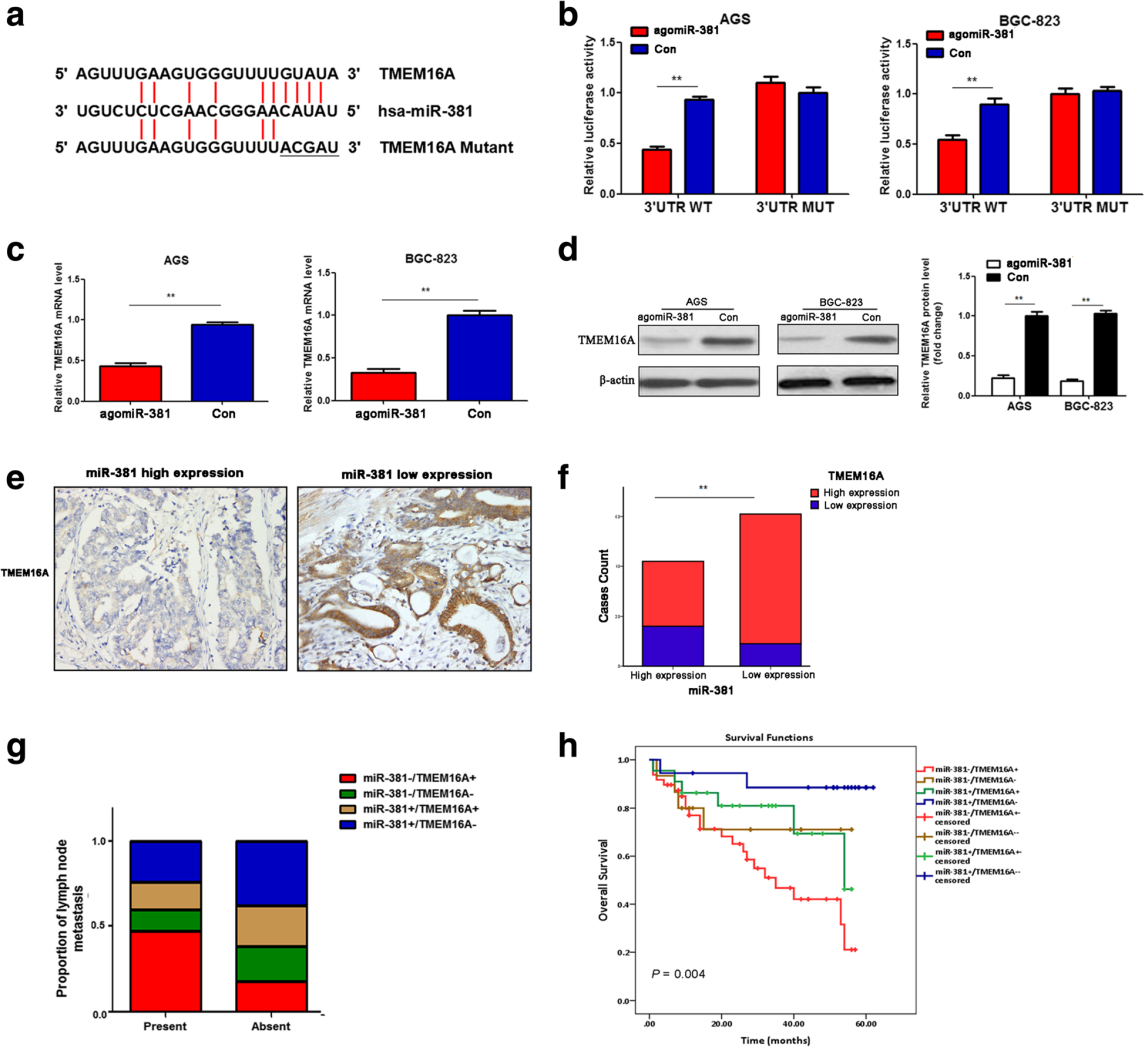
TMEM16A is a direct target of miR-381 in gastric cancer cells. **a** MiR-381 and its putative binding sequence in the wild-type and mutant 3′-UTR of TMEM16A. **b** Overexpression of miR-381 significantly decreased the luciferase activity that carried wild type (WT) but not mutant type (MUT) 3′-UTR of TMEM16A in gastric cancer cells. **P < 0.01. **c**, **d** Overexpression of miR-381 markedly suppressed the mRNA and protein levels of TMEM16A in gastric cancer cells. **P < 0.01. **e** Representative immunohistochemistry (IHC) staining of TMEM16A in high or low miR-381 expression gastric cancer tissues. **f** MiR-381 expression was inversely related to TMEM16A expression in gastric cancer tissues. **P < 0.01. **g** The correlation between miR-381/TMEM16A co-expression and the percentage of metastatic lymph nodes, *P* < 0.05. **h** Overall survival of gastric cancer patients. Patient groups were separated based on expression status of miR-381 and TMEM16A

According to bioinformatic databases (miRanda), there was a binding site of miR-381 in TMEM16A 3′-UTR (Fig. 4a). To validate that TMEM16A was a direct target gene of miR-381, luciferase assay were performed. We initially constructed two types of plasmids containing the luciferase reporting gene and wild-type or mutant TMEM16A 3′UTR (Fig. 4a) and cotransfected agomiR-381 into AGS and BGC-823 cells. Results showed that miR-381 overexpression significantly reduced wide-type TMEM16A luciferase activity, while had no inhibition effect on the mutant-type TMEM16A luciferase activity in AGS and BGC-823 cells (Fig. 4b). Next, qRT-PCR and western blot assay was performed to investigate whether the mRNA and protein expression of TMEM16A was influenced. Compared to the negative control group, both the TMEM16A mRNA and protein level was markedly down-regulated in miR-381 overexpression group (Fig. 4c and d). These results indicated that TMEM16A was a direct target of miR-381.

To further confirm that TMEM16A was negatively regulated by miR-381 in gastric cancer, we examined the expression of TMEM16A protein using immunohistochemistry in gastric cancer tissues. Compared with that in tissues of low expression of miR-381, the expression of TMEM16A potein was significantly lower in tissues with a high level of miR-381 (Fig. 4e). Moreover, the expression of miR-381 was inversely related to the level of TMEM16A expression in gastric cancer tissues (Fig. 4f, Table 2). Then, according to the expression level of miR-381 mRNA and TMEM16A protein, we diveded 103 gastric cancers into four groups, miR-381 low expression and TMEM16A high expression (miR-381-/TMEM16A+), miR-381 low expression and TMEM16A low expression (miR-381-/TMEM16A-), miR-381 high expression and TMEM16A high expression (miR-381+/TMEM16A+), miR-381 high expression and TMEM16A low expression (miR-381+/TMEM16A-), and their association with lymph node metastasis and overall survival (OS) was analyzed. The results showed that miR-381-/TMEM16A+ group was associated with a significantly higher metastasis rate (Fig. 4g). Moreover, miR-381-/TMEM16A+ predicted poor prognosis, while miR-381+/TMEM16A- indicated relative favorable prognosis (Fig. 4h). These results suggested that the increase of TMEM16A expression by inhibition of miR-381 has a critical role in promoting gastric cancer invasion and metastasis.

**Table 2:** The relationship between miR-381 expression and TMEM16A expression in gastric cancer tissues by Phiand Cramers V correlation analysis

Variables		All cases	TMEM16A		*P* value	Phi
			Low (%)	High (%)		
miR-381	Low (%)	62	9 (14.5%)	53 (85.5%)	0.005	−0.280
	High (%)	41	16 (39.1%)	25 (60.9%)		

### TMEM16A mediates the functional effects of miR-381 on migration and invasion in gastric cancer cells

**Fig. 5:**
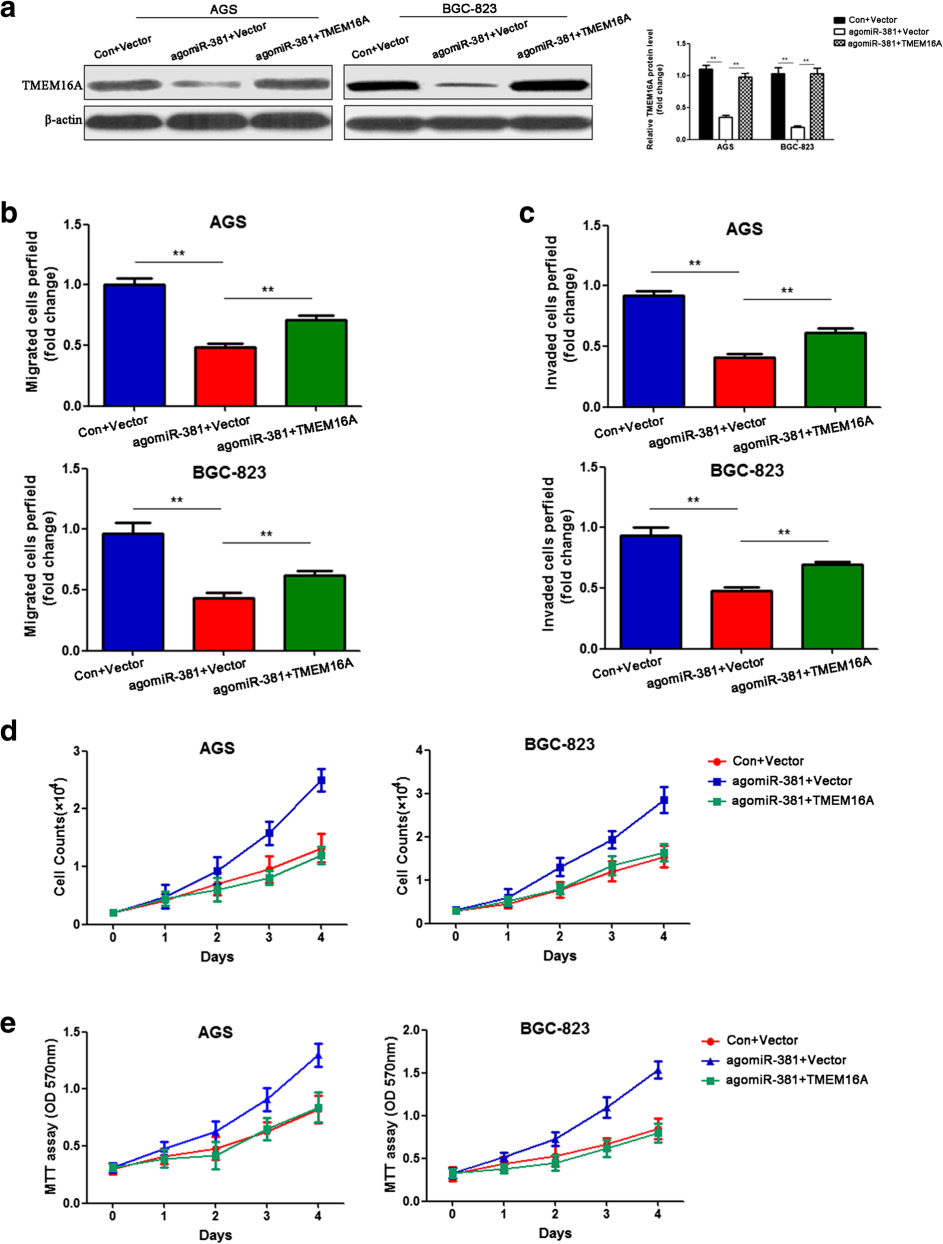
TMEM16A mediates the effects of miR-381 on migration and invasion in gastric cancer cells. Gastric cancer cells tranfected with agomiR-381 and negative control, together with blank vector or TMEM16A overexpression plasmid. **a** Western blot analysis showed that ectopic expression of miR-381 reduced the TMEM16A protein expression, while co-transfection of TMEM16A overexpression TMEM16A plasmids could recover the TMEM16A expression. **P < 0.01. **b**, **c** Co-transfection of TMEM16A overexpression reverted the suppressive effects of miR-381 overexpression on the migration and invasion of gastric cancer cells**P < 0.01. **d**, **e** TMEM16A-overexpressing did not reverse the inhibition of gastric cancer cell proliferation induced by the miR-381 overexpression

After indicating that miR-381 suppressed gastric cancer cell invasion in vitro and in vivo and identifying TMEM16A as a direct target of miR-381, we next focused on whether TMEM16A could mediate the biological function of miR-381 in gastric cancer. First, we transduced agomiR-381 or negative control and overexpression of TMEM16A plasmids. We found that ectopic expression of miR-381 reduced the TMEM16A protein expression, while co-transfection of TMEM16A overexpression TMEM16A plasmids could recover the TMEM16A expression (Fig. 5a). Furthermore, co-transfection of TMEM16A overexpression reverted the suppressive effects of miR-381 overexpression on the migration and invasion of gastric cancer cells (Fig. 5b and c). However, supplement of TMEM16A-overexpressing did not reverse the inhibition of gastric cancer cell proliferation induced by the miR-381 overexpression (Fig. 5d and e). It was consistent with our previous finding that knockdown of TMEM16A did not affect proliferation of gastric cancer cells, suggesting that miR-381 stimulated gastric cancer cell proliferation through any other targets rather than TMEM16A.

### MiR-381 suppresses TGF-β signaling pathway and down-regulates EMT phenotypes

**Fig. 6:**
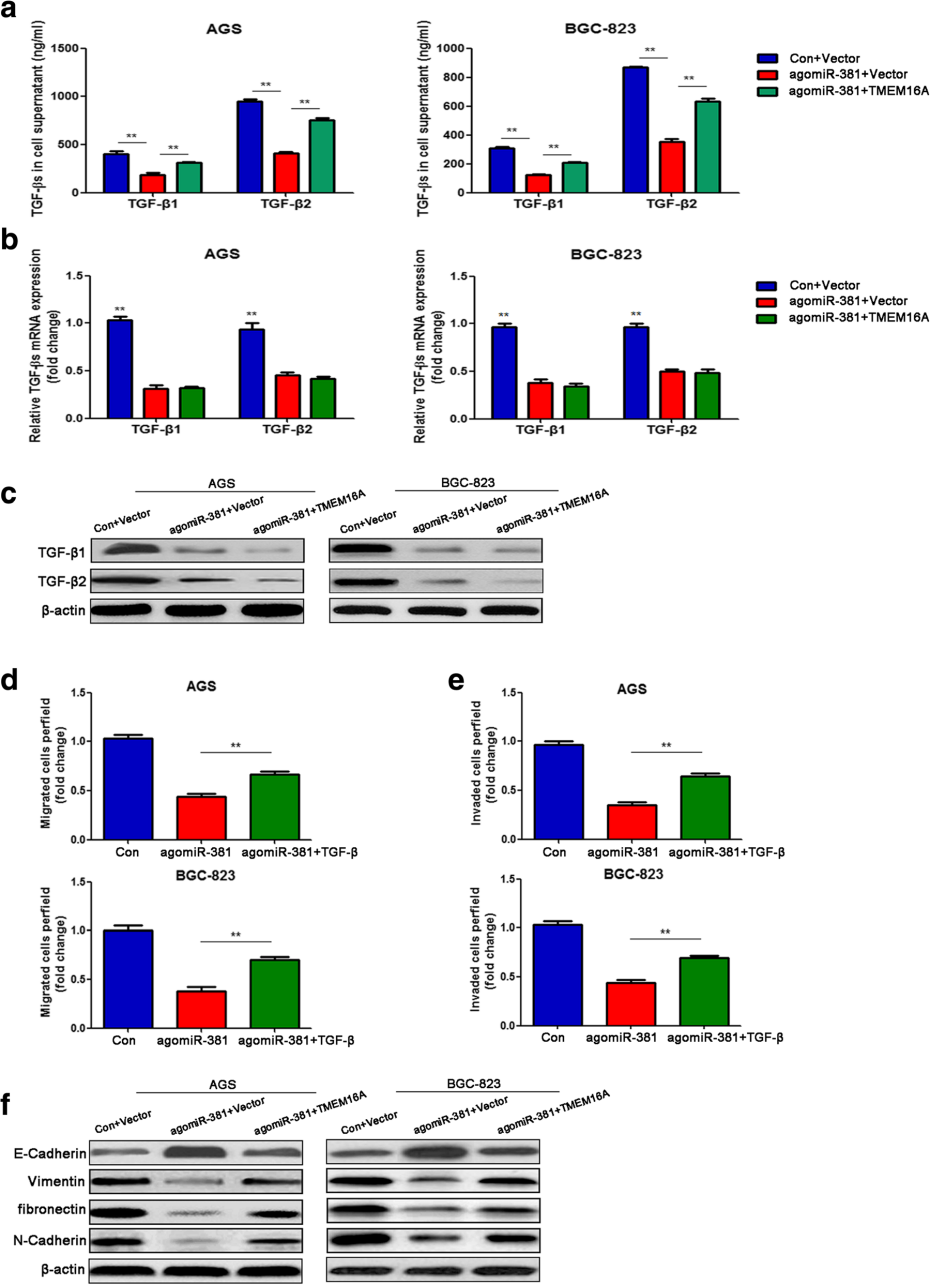
Mir-381 suppresses TGF-β signaling pathway and down-regulated EMT phenotypes. Gastric cancer cells tranfected with anomiR-381 and negative control, together with blank vector or TMEM16A overexpression plasmid. **a** ELISA assay indicated that supernatant concentrations of TGF-β1 and TGF-β2 dramatically reduced in agomiR-381 group, and overexpression of TMEM16A could partially rescue their expression. **P < 0.01. **b** QRT-PCR assay showed that mRNA of TGF-βs decreased in agomiR-381 group, which not be affected by overexpression of TMEM16A. **c** Protein expression of TGF-β1 and TGF-β2 in gastric cancer cells down-regulated in miR-381 overexpression group, and further decreased in TMEM16A overexpression group. **d**, **e** Transwell assay showed that the effect of miR-381 on gastric cancer cells migration and invasion could be partially recovered by supplement of TGF-β. **P < 0.01. **f** Western blot assay was employed to detect the expression of EMT related markers in gastric cancer cells

Our previous study found that TMEM16A contributed to gastric cancer cell invasion through promoting TGF-βs secretion [31]. Therefore, we explored whether miR-381 suppressed TGF-βs signaling pathway via targeting TMEM16A. First, cell supernatants were gathered and concentrations of TGF-β1 and TGF-β2 were measured by sandwich ELISA. Compared to negative control, miR-381 overexpression significantly reduced TGF-β1 and TGF-β2 levels, while co-transfection of TMEM16A overexpression could rescue the down-regulation of TGF-β1 and TGF-β2 levels partially (Fig. 6a). We previously reported that TMEM16A promoted TGF-βs secretion rather than synthesis, hence, we further investigated the mRNA level of TGF-βs in AGS and BGC-823 cells. Surprisingly, miR-381 overexpression could significantly abolish the mRNA expression of TGF-β1 and TGF-β2. However, overexpression of TMEM16A did not rescue the mRNA expression of TGF-βs (Fig. 6b). In protein level, TGF-β1 and TGF-β2 were significantly decreased in miR-381 overexpression group compared with the negative control group. While co-transfection of TMEM16A expression, TGF-β1 and TGF-β2 protein expression were further decreased (Fig. 6c). These data indicated that miR-381 reduced TGF-βs secretion partially through targeting TMEM16A, and miR-381 inhibited TGF-βs synthesis via other pathways rather than targeting TMEM16A. To further confirm TGF-β was involved in the suppressive effect of miR-381 on migration and invasion, recombinant purified TGF-β was added to miR-381 overexpression group. Supplement of TGF-β significantly reverted the ability of invade and migrate in gastric cancer cell which were inhibited by miR-381 overexpression (Fig. 6d and e).

Based on the fact that TGF-β can induce epithelial–mesenchymal transition (EMT) [36], and our previous finding that TMEM16A suppressed E-Cadherin expression through promoting TGF-β secretion [31], we further detected the effect of miR-381 on EMT related markers. Our results showed that miR-381 overexpression could notably down-regulate mesenchymal markers, vimentin, fibronectin, N-Cadherin, but up-regulated epithelial marker E-Cadherin, and enforced TMEM16A expression could partially rescue the expression of these EMT markers (Fig. 6f). These data demonstrated that miR-381 could inhibit TGF-β signaling pathway and down-regulate EMT phenotype partially through targeting TMEM16A, and miR-381/TMEM16A/TGF-β/EMT axis contributed to the migration and invasion of gastric cancer cells.

## Discussion

Numbers of miRNAs have been identified to be involved in a variety of tumorigenic processes, including cell proliferation, apoptosis, migration and invasion. In this study, the oncological significance of miR-381 in gastric cancer was investigated and it was demonstrated that: (1) miR-381 expression was significantly decreased in gastric cancer tissues and cell lines, (2) low expression of miR-381 was associated with lymph node metastasis, advanced tumor stage and poor prognosis of gastric cancer patients, (3) down-regulation of miR-381 contributed to gastric cancer proliferation and metastasis in vitro and in vivo, (4) TMEM16A was a direct target of miR-381 and they were inversely correlated with each other in clinical gastric cancer specimens, (5) TMEM16A mediated the functional effects of miR-381 on migration and invasion rather than proliferation of gastric cancer, (6) miR-381 acted as a suppressor gene miRNA partially through suppressing TGF-β signaling pathway and EMT.

MiR-381 was mapped to the chromosomal 14q32.31 locus where existed a cluster of miRNAs, such as miR-154 and miR-377, which have been reported to act as a tumor suppressor in several cancers [9]. For example, miR-154 was down-regulated in breast cancer and inhibited growth and invasion [37] and suppressed hepatocellular carcinoma tumorigenic and metastatic potential in vitro and in vivo [38]. MiR-377 overexpression reduced cell proliferation and suppressed invasion of osteosarcoma cells [39] and impeded the ability of clear cell renal cell carcinoma cells to proliferate, migrate and invade [40]. With regard to miR-381, it has been widely reported as a potential tumor suppressor miRNA in previous studies. Up-regulation of miR-381 expression could abrogated cancer cells proliferation, invasion and migration in various solid tumors [11–17]. Moreover, miR-381 increased the sensitivity of renal cancer cells to 5-fluorouracil (5-FU) [41] and modulated the multidrug resistance (MDR) phenotype in leukemia cells and increased their drug uptake [42]. Consistent with these studies, the present study found that miR-381 was markedly decreased in gastric cancer, and miR-381 expression prohibited gastric cancer cells proliferation, invasion and migration in vitro and in vivo and predicted favorable prognosis. However, the potential role of miR-381 as a onco-miRNA also has been uncovered. Tang et al. [18, 19] showed that miR-381 expression was increased in glioma and promoted tumor cell pathological malignant progression. Li et al. [22] found a high expression of miR-381 in osteosarcoma and the association with an inferior prognosis, and suppression of miR-381 expression increased the sensitivity of osteosarcoma cells to cisplatin. In fact, there were many miRNAs like miR-381 have been demonstrated to play both tumor-suppressing and tumor-promoting roles that depend on the cancer types. For instance, miR-377, which was also located in 14q32.31, unlike the role in osteosarcoma [39] and renal cell carcinoma [40], increased in gastric cancer and promoted cell proliferation [43]. MiR-204 has been demonstrated to have a dual function as a tumor-suppressive miRNA and/or an oncomiR in different cancers [44–46]. Even in the same cancer type, miR-204 also had a dual regulatory function in different cancer subtypes. In prostate cancer, miR-204 acted as an oncomiR in neuroendocrine-like prostate cancer cells but as a tumor suppressor in prostatic adenocarcinoma cells [47]. These findings showed the complexity of miRNAs including miR-381 in cancers, more than that, suggesting that therapies targeting miRNAs must consider their potential dual role in cancers.

In regard to the upstream regulatory mechanisms of miR-381, Liang et al. [17] reported that P53 binds to the promoter of miR-381, activating miR-381 transcription and inducing its expression. Hou et al.[48] found that the expression levels of transcriptional factors Sox9 and Runx2 are positively correlated with transcription of miR-381, indicating they may regulate expression of miR-381. However, the regulation of miR-381 has not been thoroughly studied. In deed, the regulation mechanisms of microRNA are very complicated, in that microRNA can be regulated at different levels including the pre-transcriptional, transcriptional, and post-transcriptional level [49, 50]. Such as DNA copy number variation, DNA methylation, histone modification, transcription factor (TF) and post-transcripitonal modification, which were involved in the regulation of microRNA [51–54]. Further investigations are needed to explore the regulation of miR-381 in the furture.

TMEM16A, a potential oncogene, was found to be amplified as part of human chromosome 11q13 amplicon, which may be one reason for TMEM16A overexpression [55, 56]. Our previous results showed that TMEM16A overexpression was more pervasive than amplification in gastric cancer [31], suggesting that overexpression of TMEM16A in gastric cancer may have any other regulatory mechanisms. To date, the regulation of TMEM16A remains largely unknown. In prostate cells, Cha et al. [57] found that the promoter region of TMEM16A contains putative binding sites for an androgen response element (ARE), which allow testosterone-induced TMEM16A overexpression. Signal transducer and activator of transcription 6 (STAT6) binding site was also found in TMEM16A promoter region, leading to IL-4-induced TMEM16A up-regulation [58]. In addition, epigenetic factors, such as methylation level of its promoter region [59], histone deacetylases (HDACs) [60], were reported to regulate the expression of TMEM16A. Recent study revealed that TMEM16A (ANO1) was a direct target gene of miR-132, and was negatively regulated by miR-132 in colorectal cancer [32]. One given gene might be regulated by multiple miRNAs, while one given miRNA could have various target genes [61, 62]. Consistently, in the present study, we found that miR-381 directly targeted 3′UTR of TMEM16A and negatively modulated the expression of TMEM16A in gastric cancer, moreover, enforced overexpression of TMEM16A effectively reversed the tumor suppressive functions of miR-381 on gastric cancer migration and invasion. These results confirmed that miR-381 was one of the upstream regulators of TMEM16A and by which exerted its suppressive role in gastric cancer.

**Fig. 7:**
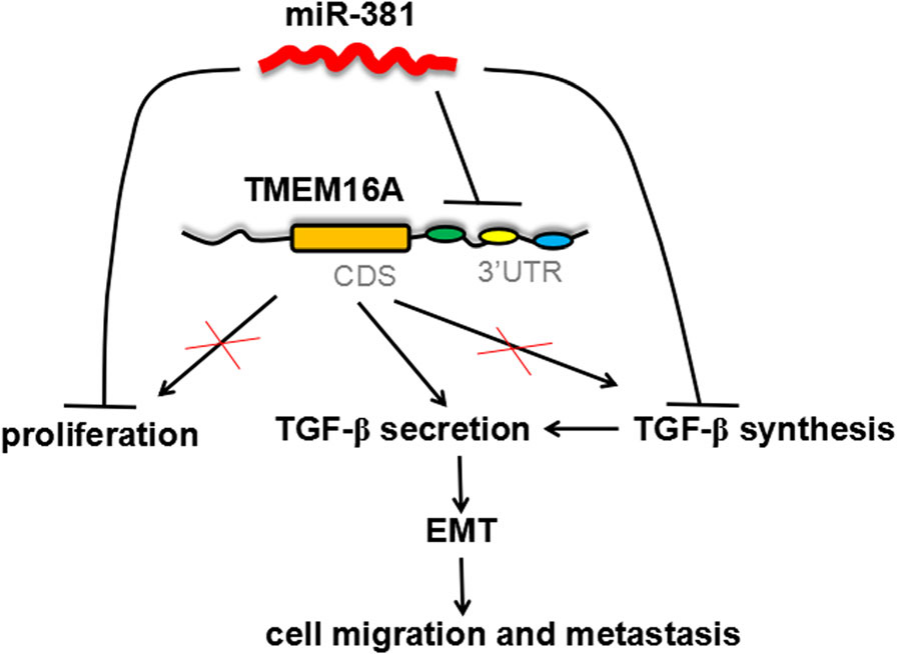
Model for miR-381-mediated inhibition of cell proliferation and migration via its regulation of the TMEM16A and TGF-β signaling pathway. *CDS* coding sequence, *3′UTR* 3′-untranslated region, *EMT* epithelial-mesenchymal transition

Our previous study found that TMEM16A facilitated gastric cancer invasion and migration through suppressing E-Cadherin expression via promoting TGF-β secretion [31]. TGF-β was the most potent and most well-described inducer for EMT [36]. After identifying TMEM16A was a direct target of miR-381, our studies further showed that miR-381 abolished TGF-β synthesis and secretion, and subsequently down-regulated the expression of EMT phenotype. Indeed, several signaling pathways were reported to be involved in the functional role of miR-381. In epithelial ovarian cancer, miR-381 targeted YY1 and regulated p53 and Wnt signaling [12]. In glioma, miR-381 increased the proliferation of tumor cells by targeting LRRC4 and this action is associated with inducing MEK/ERK and AKT signaling [18]. Inhibition of miR-381 sensitized glioblastoma cells to temozolomide (TMZ) by inhibiting the mTOR pathway through targeting NEFL [63]. In addition, miR-381 contributed to respiratory infection through increasing the activity of NF-κB signaling by directly targeting IκBα [64]. These studies indicated the complicated role and mechanism of miR-381 depending on different cancer types and molecular targets. In the present study, miR-381 did not only influence the secretion but also the synthesis of TGF-β, suggesting that other molecules or pathways than targeting TMEM16A were involved in the influence of miR-381 on TGF-β. Our data also found that miR-381 could impair the expression of EMT phenotype through miR-381/TMEM16A/TGF-β axis. However, other pathways by which miR-381 regulated EMT have been reported. For instance, Twist, an important inducer of EMT, was found to be directly targeted by miR-381 [14]. The underlying mechanism linking miR-381 and EMT should be further investigated (Fig. 7).

## Conclusions

In this study, we finds for the first time that miR-381 is decreased in gastric cancer and its down-regulation is asociated with poor clinical features of gastric cancer patients. In vitro and in vivo experiments demonstrated that miR-381 impedes gastric cancer proliferative and metastatic behaviors. Mechanistically, we confirm that miR-381 suppressed invasion and migration and EMT of gastric cancer cells by targeting TMEM16A partially through TGF-β signaling pathway (Fig. 7). Collectively, miR-381 may serve as a novel therapeutic target for treating gastric cancer.

## Additional files

Additional file 1: Figure S1. Confirmation of miR-381 overexpression in gastric cancer cells. QRT-PCR analysis of miR-381 transfection efficiency after agomiR-381 and negative control transfection in AGS and BGC-823 cell lines. (TIF 31 kb)
Additional file 2: Figure S2. Confirmation of miR-381 low-expression in gastric cancer cells. QRT-PCR analysis of miR-381 transfection efficiency after antagomiR-381 and negative control transfection in MKN-28 and SGC-7901 cell lines. (TIF 29 kb)

## Abbreviations

3′UTR: 3′-untranslated region
ELISA: Enzyme-linked immunosorbent assay
EMT: Epithelial-mesenchymal transition
GEO: Gene expression omnibus
IHC: Immunohistochemistry
MDR: Multidrug resistance
MiR-381: microRNA-381
OS: Overall survival
PFS: Progression-free survival
TGF-β: Ransforming growth factor beta
TMEM16A: Transmembrane protein 16A
