# Supplementary figures and images for: MicroRNA-381 inhibits the metastasis of gastric cancer by targeting TMEM16A expression
Source: J Exp Clin Cancer Res. 2017 Feb 13;36:29. doi: 10.1186/s13046-017-0499-z (PMC5307754; doi:10.1186/s13046-017-0499-z)

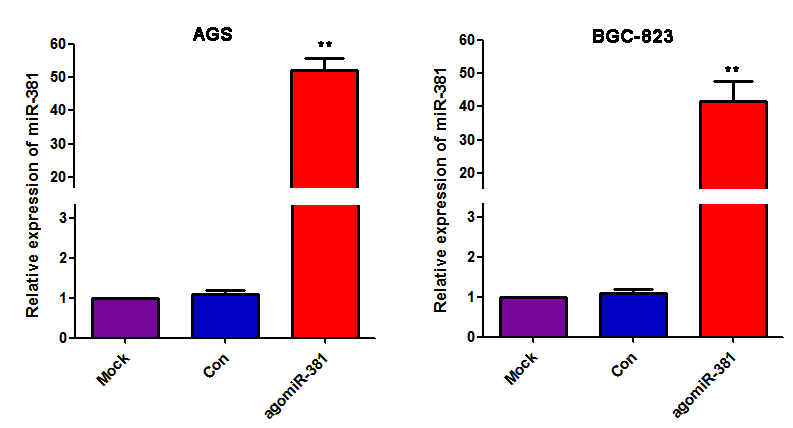

Supplement: Additional file 1: Figure S1. — Confirmation of miR-381 overexpression in gastric cancer cells. QRT-PCR analysis of miR-381 transfection efficiency after agomiR-381 and negative control transfection in AGS and BGC-823 cell lines. (TIF 31 kb) [file 13046_2017_499_MOESM1_ESM.tif]

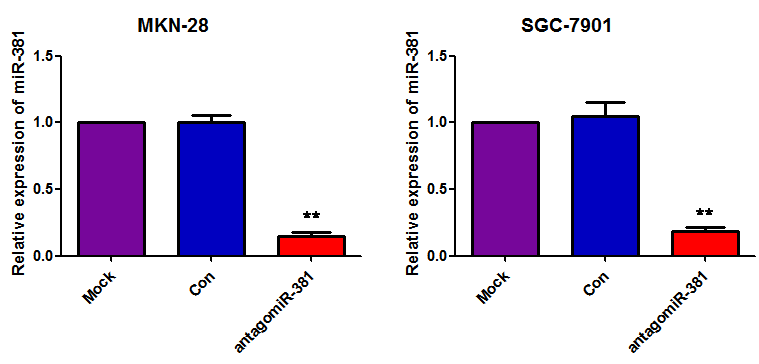

Supplement: Additional file 2: Figure S2. — Confirmation of miR-381 low-expression in gastric cancer cells. QRT-PCR analysis of miR-381 transfection efficiency after antagomiR-381 and negative control transfection in MKN-28 and SGC-7901 cell lines. (TIF 29 kb) [file 13046_2017_499_MOESM2_ESM.tif]
